# Optimization of a Coupled Neuron Model Based on Deep Reinforcement Learning and Application of the Model in Bearing Fault Diagnosis

**DOI:** 10.3390/s25123654

**Published:** 2025-06-11

**Authors:** Shan Wang, Jiaxiang Li, Xinsheng Xu, Ruiqi Wu, Yuhang Qiu, Xuwen Chen, Zijian Qiao

**Affiliations:** 1 Tianjin Key Laboratory for Advanced Mechatronic System Design and Intelligent Control, School of Mechanical Engineering, Tianjin University of Technology, Tianjin 300384, China; 15900226087@163.com (S.W.); 13123747676@163.com (J.L.); 17861825276@163.com (X.X.); reiky@stud.tjut.edu.cn (R.W.); 13694300332@163.com (Y.Q.); 2 National Demonstration Center for Experimental Mechanical and Electrical Engineering Education, Tianjin University of Technology, Tianjin 300384, China; 3 Zhejiang Pumai Technology Co., Ltd., Hangzhou 315000, China; chenxuwen@premax.com.cn; 4 Faculty of Mechanical Engineering and Mechanics, Ningbo Key Laboratory of Micro-Nano Motion and Intelligent Control, Ningbo University, Ningbo 315211, China; 5 Department of Mechanical and Aerospace Engineering, The Hong Kong University of Science and Technology, Hong Kong SAR, China

**Keywords:** coupled neuron, deep reinforcement learning, parameter optimization, fault diagnosis

## Abstract

Bearings are critical yet vulnerable components in mechanical equipment, with potential failures that can significantly impact system performance. As stochastic resonance methods effectively convert noise energy into fault characteristic energy within bearing vibration signals, they remain a research focus in bearing fault diagnosis. This study proposes a coupled neuron model based on biological stochastic resonance effects for processing bearing vibration signals. To enhance parameter optimization, we develop an improved deep reinforcement learning algorithm that incorporates a prioritized experience replay buffer into the network architecture. Using the SNR as the evaluation metric, the algorithm performs data screening on the replay buffer parameters before training the deep network for predicting coupled neuron model performance. In terms of experimental content, the study performed data processing on simulated signals and vibration signals of gearbox bearing faults collected in the laboratory environment. By comparing the coupled neuron model optimized with a reinforcement learning algorithm, particle swarm algorithm, and quantum particle swarm algorithm, the experimental results show that the coupled neuron model optimized with a deep reinforcement learning algorithm has the optimal signal-to-noise ratio of the output signal and recognition rate of the bearing faults, which are −13.0407 dB and 100%, respectively. The method shows significant performance advantages in realizing the energy enhancement of the bearing fault eigenfrequency and provides a more efficient and accurate solution for bearing fault diagnosis, which has important engineering application value.

## 1. Introduction

As core components of mechanical equipment such as fans, pumps, and compressors in rotating machinery, bearings perform critical functions in load-bearing and power transmission [1,2,3]. Their health conditions directly affect operational stability and safety, and the service life of equipment [4,5,6]. Bearing fault diagnosis not only concerns equipment reliability and economic costs, but also serves as a vital foundation for production safety and technological iteration [7,8,9,10].

Early diagnosis primarily relies on expert experience and time-frequency analysis tools, which suffer from strong subjectivity and low feature extraction efficiency [11,12,13]. Traditional methods exhibit insufficient robustness under variable operating conditions and strong noise environments [14,15], while also struggling with high-dimensional nonlinear data processing. In industrial scenarios where fault samples are scarce and imbalanced in distribution, conventional models are prone to overfitting or underfitting [16,17,18]. Gruber et al. [19] used the fast Fourier transform (FFT) of a broadband accelerometer to calculate the spectral content of rolling bearing vibration signals. Rodriguez et al. [20] proposed a rolling bearing fault diagnosis method, which combines the Extreme Learning Machine (ELM) algorithm, the Static Wavelet Transform (SWT), and the Singular Value Decomposition (SVD) with high diagnostic accuracy under variable speed conditions. Li, Xin et al. [21] developed a constant speed rolling bearing fault diagnosis method based on Variable Mode Decomposition–Fractional Fourier Transform (VMD-FRFT), which provides an effective filtering algorithm for fundamental frequency extraction and instantaneous frequency multiplication. Although traditional methods have achieved progress in fault diagnosis, their inherent processing limitations reveal a gap between theoretical frameworks and practical industrial scenarios. In contrast, biological neurons, through spike encoding mechanisms, not only enable efficient feature extraction from complex multimodal signals but also demonstrate strong adaptability to noisy environments and dynamic working conditions [22].

Neurons achieve information transmission through electrochemical signals, namely action potentials and synaptic connections, a mechanism that reveals their high efficiency in signal selection and integration. Artificial sensory neurons can simultaneously perform signal perception and spike encoding, significantly enhancing the efficiency and accuracy of fault diagnosis [23]. Panpan Guo et al. [24] proposed a novel adaptive gated neuron with physical feature weighting, theoretically demonstrating its superior feature extraction capability. This method enables efficient and reliable bearing fault diagnosis under strong noise interference. He, Lifang et al. [25] developed a high-dimensional coupling system based on the FitzHugh–Nagumo (FHN) neuron model for bearing fault diagnosis, improving diagnostic reliability and accuracy across diverse applications. Liao, Jingxiao et al. [26] introduced a model comprising quadratic neurons, which effectively constrains noisy data through enhanced feature representation capabilities. While research on neuronal applications in signal and image processing demonstrates deep integration between biological mechanisms and artificial systems, neuron models exhibit inherent limitations, including noise sensitivity [27], unstable diagnostic accuracy under noisy signals, and weak generalization capabilities. Optimizing parameters in neuron models can substantially improve their performance, thereby advancing precision and speed in bearing fault detection [28].

Deep reinforcement learning (DRL) dynamically adjusts optimization strategies through agent–environment interactions based on real-time feedback, demonstrating exceptional effectiveness in optimizing neuron models [29,30]. As a DRL implementation, Deep Q-Learning (DQL) builds upon traditional Q-Learning rooted in the Markov Decision Process (MDP), which iteratively updates action–value functions via Bellman equations. However, traditional Q-Learning struggles with high-dimensional state spaces [31,32]. The tabular Q-value storage mechanism fails to process complex inputs, while the emergence of deep neural networks addresses high-dimensional challenges through end-to-end feature extraction [33,34], replacing manual feature engineering in conventional methods [35,36]. This advancement establishes the generalization capability foundation for Q-Learning [37,38,39]. Chen, Cheng et al. [40] proposed an enhanced DRL algorithm whose simulations achieved maximum rewards in both static and complex environments, exhibiting optimal convergence with the minimal average steps and shortest runtime for target localization. Kang, Yuxiang et al. [41] developed a dual-input anomaly detection method based on DRL and validated its efficacy in fault detection for real aircraft engine rolling bearings. The success of DRL confirms the potential of autonomous learning through environmental interactions, where discounted reward mechanisms optimize cumulative multi-step decision returns and resolve parameter adjustment latency [42]. Nevertheless, DRL still suffers from Q-value overestimation issues, particularly in large action spaces, leading to training instability, ineffective network learning with low convergence rates, and susceptibility to local optima [43,44].

Aiming at the above problems, this paper proposes an SNR-based empirical playback method for improving the DQL algorithm, which innovatively integrates the signal-to-noise ratio difference analysis with the principle of stochastic resonance, and employs a coupled neuron model for noise-assisted enhancement of bearing vibration signals. In the deep reinforcement learning framework, the priority experience playback mechanism is innovatively combined with the SNR optimization objective, the playback area data is screened by taking the signal-to-noise ratio as the optimization objective, and the coupled model parameters are predictively trained by combining with the deep network, which ultimately forms the deep reinforcement learning-driven adaptive parameter optimization algorithm, so as to improve the recognition accuracy of the characteristic frequency of bearing faults. The method can accelerate the convergence and improve the data utilization, so that the network can reach the convergence state faster and reduce the training time, and thus the optimal parameter combination of the coupled neurons can be obtained faster. Through the processing of simulation signals and laboratory measurement of gearbox bearing fault vibration signals, this paper constructs a coupled neuron optimization model based on deep reinforcement learning. By comparing and analyzing the performance of similar models optimized by a reinforcement learning algorithm, particle swarm algorithm, and quantum particle swarm algorithm, the experimental results show the following: the coupled neuron model optimized by a deep reinforcement learning algorithm shows the optimal performance in terms of signal-to-noise ratio index improvement and fault feature recognition accuracy, with 100% accuracy of fault feature recognition, and the gain of SNR reaches −13.0407 dB (compared with the increase of 0.4321 dB in QPSO), which verifies the effectiveness of the method in bearing fault diagnosis.

The remainder of this paper is organized as follows. Section 2 presents the theoretical framework, detailing how the deep reinforcement learning architecture integrates SNR-optimized data screening from the replay buffer with deep network-based training of coupled model parameters, thereby establishing the theoretical foundation for the optimization algorithm. Section 3 describes the simulation study, including dynamic characteristic modeling of rolling bearing motion and comparative analysis with alternative algorithms. Section 4 validates the performance advantages of the DRL-optimized coupled neuron model through experimental evaluations of output signals and bearing fault diagnosis. Section 5 summarizes the theoretical and experimental findings, followed by a discussion of future research directions.

## 2. Theory

This section employs a coupled neuron model within a nonlinear system framework, utilizing SNR as the evaluation metric to perform data screening on training experiences from the prioritized experience replay buffer. The filtered data is subsequently fed into a deep network for predictive training of coupled neuron model performance, enabling adaptive parameter optimization of the enhanced DRL algorithm for driving the coupled neuron system.

### 2.1. Coupled Neuron

The coupled neurons of a nonlinear system can be represented as follows [45]:

(1) $$\left\{\begin{array}{c} \frac{dx}{dt}\;=-wf\;x+\lambda tanhx+\delta (y-x)+in(t)\\ \frac{dy}{dt}\;=-ay+\frac{2b}{R}\;yexp(-\frac{y^{2}}{k^{2}}\;)+\delta (y-x) \end{array}\right.$$

The coupling strength is *δ*, *δ* ∈ [−1,1], which is used to control the interaction strength between two neurons. The hyperbolic tangent neuron function in the coupled neuron is as follows:

(2) $$U_{1}\;(x)=\frac{w_{f}\;x^{2}}{2}-\lambda ln(coshx)$$

The parameter *w_f_* > 0 represents the coefficient of the quadratic term, and *λ* > 0 denotes the coefficient of the logarithmic term. The adjustment of *w_f_* and *λ* enables switching between monostable and bistable states. The Gaussian neuronal function is expressed as follows:

(3) $$U_{2}\;(x)=\frac{ax^{2}}{2}\;+bexp(-\frac{x^{2}}{R^{2}}\;)$$

where *a* > 0 is the quadratic coefficient, *b* > 0 represents the exponential decay rate, and *R* > 0 represents the scale factor. By adjusting *a*, *b*, and *R*, the switching between the monostable state and the bistable state can be achieved. Noise input in coupled neurons is defined as *in*(*t*), and the formula is as follows:

(4) $$in(t)=A_{0}\;cos(\Omega t)+\sqrt{2D}\;\xi (t)$$

In the formula, parameter *A*_0_ is the amplitude of the periodic signal to be detected, Ω is the angular frequency of the periodic signal to be detected, *D* is the intensity of Gaussian white noise, and *ξ*(*t*) is the standard white Gaussian noise process.

The variables *x* and *y* in the coupled neuron represent the state evolution trajectories of the hyperbolic tangent neuron and the Gaussian neuron, respectively. Each neuron realizes dynamic matching between weak signal detection and the steady-state state through the bistable characteristic. The coupling mechanism *δ*(*y – x*) promotes the two neurons to work together, thereby enhancing the system’s sensitivity to periodic signals [46]. The coupling mechanism of neurons is that when *δ* > 0, the system tends to a synchronous state, and when *δ* < 0, it tends to an asynchronous state. By adjusting the phase relationship between *δ* and the external stimulus *A*_0_, filtering and enhancement of specific frequency signals can be achieved.

In coupled neuron models, the SNR can be optimized by adjusting the parameters *w_f_*, *λ*, *a*, and *b*. The SNR serves as a core metric for evaluating signal quality by quantifying the proportional relationship between signal and background noise. It is formally defined as the ratio of signal power (or intensity) to noise power (or intensity), expressed mathematically as follows [47]:

(5) $$SNR\;\left(dB\right)=10log_{10}\;(\frac{P_{signal}}{P_{noise}\;}\;\;)$$

Among them, *P_signal_* and *P_noise_* are the powers of signal and noise, respectively. In the coupled neuron model, a high signal-to-noise ratio means that the signal is clearer and the noise interference is less, while a low signal-to-noise ratio may affect system performance.

### 2.2. DRL Algorithm

Reinforcement learning (RL) is a method of machine learning where an agent learns optimal behavioral policies through environmental interactions by maximizing long-term cumulative rewards based on feedback signals. The agent’s action–selection rule, termed the policy, defines a mapping from states to actions to identify the optimal policy [48].

Q-Learning is a form of RL algorithm implementation based on value iteration, designed to learn optimal policies by estimating Q-values (quality functions) for state–action pairs. The Q-function *Q*(*s*, *a*) quantifies the expected reward for performing action *a* in state *s*. Initially, Q-values are stored in a Q-table (rows: states; columns: actions), initialized to zeros or random values.

The Bellman equation is the mathematical basis for Q-Learning, and the formula is as follows [49]:

(6) $$Q(s,a)\leftarrow Q(s,a)+\alpha [r+\gamma a^{\prime }max\;Q(s^{\prime },a^{\prime })-Q(s,a)]$$

which contains the TD error:

(7) $$\delta_{t}\;=R_{t}+1\;+\gamma a^{\prime }max\;Q(S_{t+1}\;,a^{\prime })-Q(S_{t}\;,A_{t\;})$$

where *γ* is the discount factor (weighing off immediate and future rewards), *r* is the immediate reward, *s*’ is the next state, *maxQ*(*S_t_*_+1_, *a*’) represents the maximum Q-value of all possible actions in the next state *S_t_*_+1_, and represents the prediction of the optimal path in the future *Q*(*S_t_*, *A_t_*), which is the estimate of the Q-value of the current state–action pair.

Q-Learning drives the iterative optimization of the Q-table through TD error. The update rules for Q-values are as follows [50]:

(8) $$Q(S_{t}\;,A_{t}\;)=Q(S_{t}\;,A_{t}\;)+\alpha \delta_{t}\;$$

where *α* is the learning rate and is used to control the update pace.

The balance between exploration and exploitation is achieved by employing the ε-greedy strategy [51]: exploring new actions randomly with probability ε to avoid local optima. Typically, ε gradually decays during training, emphasizing exploration in early stages and exploitation in later phases. Starting from the current environmental state *s*, an action *a* is selected based on the ε-greedy policy. After executing the action, the reward *r* and new state *s*’ are observed. The Q-value is then updated by adjusting *Q*(*s*, *a*) through the Bellman equation. The process iterates by transitioning to state *s*’ and repeating until the termination conditions are met. The convergence criterion is satisfied when Q-table changes stabilize (or a predefined number of training episodes is reached), at which point training terminates.

Deep reinforcement learning significantly enhances traditional Q-Learning by integrating deep neural networks with its core principles, enabling effective handling of high-dimensional state spaces while improving learning efficiency and stability [52]. Traditional Q-Learning stores Q-values for state–action pairs in a table, but encounters storage and computational bottlenecks in high-dimensional or continuous state spaces. DRL addresses the curse of dimensionality by replacing the Q-table with neural networks. In DRL, the Q-value function is parameterized as a deep neural network, which approximates the long-term expected return of state–action pairs. The Q-value function of DRL is defined as follows [53]:

(9) $$Q(s,a;\theta )=Q^{*}(s,a)$$

*Q*(*s*, *a*; *θ*) is expressed as a Q-function approximated by neural network parameters *θ*. The input is the state *s* and the output is the Q-value of all actions. Q^*^(*s*, *a*) is the theoretical optimal Q-value. The role is to process high-dimensional states through deep neural networks and solve the dimensional limitation problem of traditional Q-tables.

In the Q-Learning update formula, the target Q-value and current Q-value share the same update mechanism, leading to frequent fluctuations in target values. To address this, DRL employs a dual-network architecture: the online network updates the policy, while the target network maintains fixed parameters. The target network parameters are periodically synchronized with the online network and are used to compute the target Q-value, which stabilizes the training by reducing fluctuations in the target value. The specific formula is as follows [54]:

(10) $$TargetQ=\left\{\begin{array}{c} r_{t}\;,\\ r_{t}\;+\gamma maxa^{\prime }\;Q(s_{t+1}\;,a^{\prime };\theta^{-}), \end{array}\right.\;\begin{array}{c} done=True \end{array}$$

This separation reduces the volatility of target values and makes training more stable. For example, when calculating TD targets, the target network provides stable estimates of *Q*(*s*’, *a*’), which suppresses the propagation of instability and reduces the risk of divergence, making training more stable [55]. The loss function, also known as the mean square error, is used to calculate the square of the time-series difference (TD) error for each sample in the batch and take the expectation. The loss function is specifically expressed as follows [56]:

(11) $$L(\theta )=E(s,a,r,s^{\prime })∼D\;[{\left(TargetQ-Q(s,a;\theta )\right)}^{2}]$$

The online network *Q* (*s*, *a*; *θ*) predicts the current Q-value, the target network *Q* (*s*, *a*; *θ*^−^) provides a stable target Q-value, the parameter *θ*^−^ is periodically synchronized from *θ*, and the online network parameters are optimized through random gradient descent. Experience playback solves data-related problems in DRL training by storing and randomly sampling historical experience, and further stabilizes Q-value estimation in conjunction with the target network [57]. These mechanisms together improve the learning efficiency and stability of DRL in complex environments [58].

To optimize the experience sampling strategy for improved learning efficiency and convergence speed, this paper proposes a novel method that filters replay buffer data using the SNR as the optimization objective. Traditional experience replay employs uniform sampling, but different experiences contribute unevenly to learning effectiveness. By prioritizing experiences based on their importance, measured through SNR differences, this method establishes a refined experience replay buffer. The core idea involves filtering experiences based on their significance, ensuring that samples with substantial SNR improvements are reused more frequently to accelerate model convergence.

**Figure 1:**
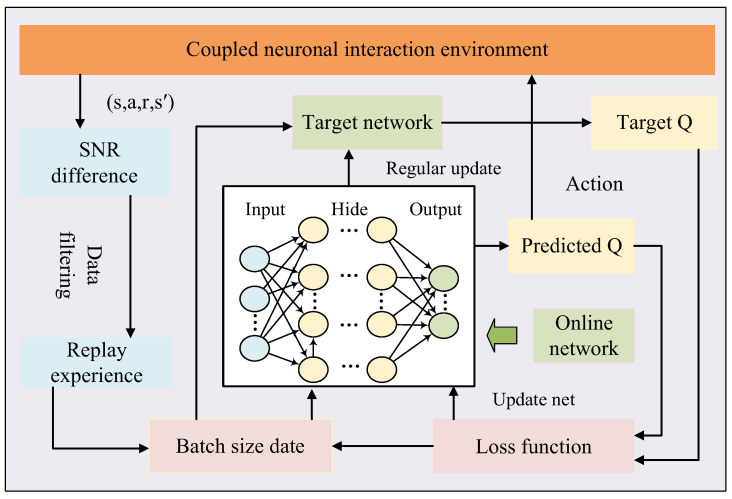
Deep reinforcement learning training methods.

In coupled neurons, the SNR difference reflects the learning efficiency of transitioning from state *S* to its successor *S* + 1. Thus, it serves as a metric to quantify the importance of each experience. A larger SNR difference corresponds to a higher priority level, as it indicates a greater contribution to improving the current policy. Here, *D_t_* represents the SNR difference of a neuron transitioning from state *S* to *S* + 1, dynamically guiding the prioritization of critical experiences in the replay buffer. The SNR difference is defined as follows:

(12) $$D_{t}\;=SNR(s_{t+1}\;,a^{\prime })-SNR(s_{t}\;,a_{t})$$

where *SNR*(*s_t_*_+1_, *a*’) is the signal-to-noise ratio value of the neuron after performing the *a*’ action in the *S_t_*_+1_ state, and *SNR*(*s_t_*, *a_t_*) is the signal-to-noise ratio value of the neuron after performing the *a_t_* action in the *S_t_* state. The core of the optimization method is to filter the playback area data through the SNR difference from noise. The deep reinforcement learning training methods designed in this paper are shown in Figure 1:

As illustrated in the figure above, the process unfolds as follows: First, the agent continuously interacts with the environment, predicting the next action via the online network and collecting training experiences. These experiences undergo SNR difference computation and data filtering before being stored in the experience replay buffer. Once sufficient data accumulates in the buffer, a batch of data is sampled. The online network computes the predicted Q-values, while the target Q-network calculates the target Q-values. The deep network is then trained to update Q-values by minimizing the loss function through gradient descent. After a predefined number of iterations, the parameters of the online network are copied to the target Q-network to synchronize their weights.

### 2.3. System Flow Design

**Figure 2:**
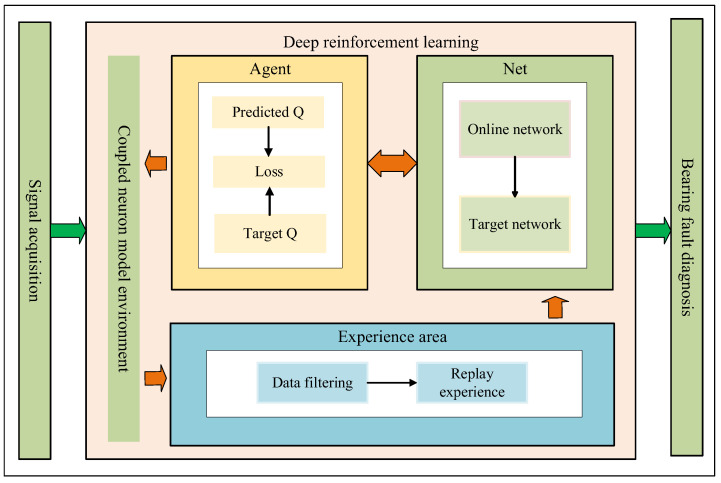
System flow design schematic diagram.

In coupled neuron models, the SNR serves as a core metric for quantifying the proportion between the signal and background noise, reflecting signal quality. A high SNR indicates clearer signals with reduced noise interference, while a low SNR may degrade system performance. To address this, this paper proposes an SNR-based experience replay method, which enables the DRL algorithm to achieve convergence more efficiently. Leveraging the improved algorithm, we optimize the parameters of the coupled neuron model to obtain enhanced model parameters and SNR levels. These advancements are subsequently applied to bearing fault detection, demonstrating improved diagnostic accuracy and robustness. Figure 2 shows the schematic diagram of this method, as follows:

- Information gathering: To address the operational specifics of bearings, sensors are strategically placed at critical locations to record bearing fault signals, ensuring data accuracy and reliability. These signals are subsequently fed into coupled neurons to extract parameter information from the neuron model. Initial training is then conducted, where the collected data is used to initialize the policy network. This initialization accelerates the transition of the initial network to a stable operational state, enabling rapid convergence and robust performance in subsequent training phases.
- Establish an experience playback area: The pre-trained optimal data is utilized as the initial state of the coupled neurons for further optimization. After the agent selects an action, the first training experience comprising the current state, action, reward, and next state is generated. The SNR difference for each state is then calculated. Using the SNR as the evaluation metric, the training experiences are filtered before being stored in the experience replay buffer. The system checks whether the number of accumulated experiences meets the minimum training batch size. If not, the agent continues to select actions and interact with the neurons to collect subsequent training data. This iterative process repeats until the experience replay buffer contains sufficient data to fulfill the minimum batch requirement, ensuring stable and efficient training initialization.
- Train the network to get the optimal parameters: Once the experience replay buffer accumulates sufficient training data, the network training process begins. A mini-batch of data is sampled from the buffer, and the online network computes predicted Q-values for these experiences. The target Q-network is then used to calculate target Q-values. The loss function derived from these values is minimized via backpropagation, and gradient descent is applied to update the weight parameters of the online network. After a predefined number of training iterations, the parameters of the online network are copied to the target Q-network, effectively creating a deep duplicate of the online network at periodic intervals to stabilize training. Finally, the system checks if the predefined number of training iterations is reached. If not, the agent selects the action with the highest Q-value (predicted by the online network) for the current state, continuing the cycle of interaction, experience collection, and network refinement until convergence criteria are met.
- Parameter output and troubleshooting: If the training iterations meet the predefined target, the optimal parameter set and the highest achievable SNR are output and integrated into the coupled neuron model for fault diagnosis. Subsequently, advanced spectral analysis techniques are applied to deeply extract characteristic frequencies in bearing signals that correlate strongly with fault patterns. This approach not only significantly enhances diagnostic efficiency but also improves the accuracy of fault detection. Furthermore, by enabling early-stage fault identification and intervention, the method drastically reduces equipment downtime. Such capabilities hold immeasurable value for ensuring production continuity and operational efficiency in industrial settings.

## 3. Simulation Illustration

The accuracy of fault diagnosis analysis based on vibration simulation data of rolling bearings largely depends on the accuracy of the dynamic model [59,60]. A single disc symmetric rotor is taken as the research object to investigate the dynamic characteristics of rolling bearings during the motion process, and a dynamic model of rolling bearings in the rotor system is constructed. In the dynamic model of rolling bearings in a rotor system, the expressions for the system kinetic energy *T*, system potential energy *U*, and dissipation function *D_f_* are as follows [61,62]:

(13) $$\;\left\{\begin{array}{l} T=\frac{1}{2}m_{R}{{\overset{\cdot }{X}}_{R}}^{2}+\frac{1}{2}m_{R}{{\overset{\cdot }{Y}}_{R}}^{2}+\frac{1}{2}m_{r}{\left({\overset{\cdot }{X}}_{r}-e\omega \sin\theta \right)}^{2}+\frac{1}{2}m_{r}{\left({\overset{\cdot }{Y}}_{r}+e\omega \cos\theta \right)}^{2}\\ \;\;\;\;\;\;\;\;\;\;\;+\frac{1}{2}m_{L}{{\overset{\cdot }{X}}_{L}}^{2}+\frac{1}{2}m_{L}{{\overset{\cdot }{Y}}_{L}}^{2},\\ U=\frac{1}{2}k_{x}{\left(X_{r}-X_{R}\right)}^{2}+\frac{1}{2}k_{x}{\left(X_{r}-X_{L}\right)}^{2}+\frac{1}{2}k_{y}{\left(Y_{r}-Y_{R}\right)}^{2}+\frac{1}{2}k_{y}{\left(Y_{r}-Y_{L}\right)}^{2},\\ Df=\frac{1}{2}c_{R}{\overset{\cdot }{X}}_{R}^{2}+\frac{1}{2}c_{R}{\overset{\cdot }{Y}}_{R}^{2}+\frac{1}{2}c_{r}{{\overset{\cdot }{X}}_{r}}^{2}+\frac{1}{2}c_{r}{{\overset{\cdot }{Y}}_{r}}^{2}+\frac{1}{2}c_{L}{{\overset{\cdot }{X}}_{L}}^{2}+\frac{1}{2}c_{L}{{\overset{\cdot }{Y}}_{L}}^{2}. \end{array}\right.$$

The equivalent stiffness of bearings 1 and 2 are $k_{R}$ and $k_{L}$, respectively, and the equivalent damping of bearings 1 and 2 are $c_{R}$ and $c_{L}$, respectively. The centrifugal force of eccentric mass during rotor rotation is $m_{r}e\omega^{2}$, where e is the mass eccentricity of the rotor [63]. The rotor and bearings are connected by equivalent stiffness $k_{r}$ and equivalent damping $c_{r}$. The reaction force of the bearing is represented by the damping force and stiffness force [64]. The centrifugal force of eccentric mass can be decomposed into *X* and *Y* direction components, which are $m_{r}e\omega^{2}\cos\theta$ and $m_{r}e\omega^{2}\cos\theta$, respectively. The rotor system is running at a speed of 1800 revolutions per minute (rpm), and the sampling frequency of *f*_s_ = 10 kHz is employed for data acquisition with a sampling time of *t* = 2 s.

Based on Newton’s second law, a dynamic model of rolling bearings in a rotor system is constructed. The description of the multi-degree of freedom dynamic model for rolling bearings under unknown time-varying noise can be expressed as follows:

(14) $$\left\{\begin{array}{l} m_{r}{\overset{..}{X}}_{r}+c_{r}{\overset{.}{X}}_{r}+k_{r}(X_{r}-(X_{R\mathrm{in}}-X_{R\mathrm{out}}))+k_{r}(X_{r}-(X_{\mathrm{Lin}}-X_{\mathrm{Lout}}))=m_{r}e\omega^{2}\cos\omega t+F_{Xr},\\ m_{r}{\overset{..}{Y}}_{r}+c_{r}{\overset{\;.}{Y}}_{r}+k_{r}(Y_{r}-(Y_{\mathrm{Rin}}-Y_{\mathrm{Rout}}))+k_{r}(Y_{r}-(Y_{\mathrm{Lin}}-Y_{\mathrm{Lout}}))=m_{r}e\omega^{2}s\mathrm{in}\omega t-m_{r}g+F_{Yr},\\ m_{\mathrm{Rin}}{\overset{..}{X}}_{\mathrm{Rin}}+c_{\mathrm{Rin}}{\overset{.}{X}}_{\mathrm{Rin}}+k_{\mathrm{Rin}}X_{\mathrm{Rin}}=-F_{X_{R}}+m_{\mathrm{Rin}}e\omega^{2}\cos\omega t,\\ m_{\mathrm{Rin}}{\overset{..}{Y}}_{\mathrm{Rin}}+c_{\mathrm{Rin}}{\overset{\;.}{Y}}_{\mathrm{Rin}}+k_{\mathrm{Rin}}Y_{\mathrm{Rin}}=m_{\mathrm{Rin}}e\omega^{2}s\mathrm{in}\omega t+m_{\mathrm{Rin}}g-F_{Y_{R}},\\ m_{\mathrm{Rout}}{\overset{..}{X}}_{\mathrm{Rout}}+c_{\mathrm{Rout}}{\overset{.}{X}}_{\mathrm{Rout}}+kX_{\mathrm{Rout}}=F_{X_{R}},\\ m_{\mathrm{Rout}}{\overset{..}{Y}}_{\mathrm{Rout}}+c_{\mathrm{Rout}}{\overset{\;.}{Y}}_{\mathrm{Rout}}+k_{\mathrm{Rout}}Y_{\mathrm{Rout}}=F_{Y_{R}}+m_{\mathrm{Rout}}g,\\ m_{\mathrm{Lin}}{\overset{..}{X}}_{\mathrm{Lin}}+c_{\mathrm{Lin}}{\overset{.}{X}}_{\mathrm{Lin}}+k_{\mathrm{Lin}}X_{\mathrm{Lin}}=-F_{X_{L}}+m_{\mathrm{Lin}}e\omega^{2}\cos\omega t,\\ m_{\mathrm{Lin}}{\overset{..}{Y}}_{\mathrm{Lin}}+c_{\mathrm{Lin}}{\overset{\;.}{Y}}_{\mathrm{Lin}}+k_{\mathrm{Lin}}Y_{\mathrm{Lin}}=m_{\mathrm{Lin}}e\omega^{2}s\mathrm{in}\omega t+m_{\mathrm{Lin}}g-F_{Y_{\mathrm{Lin}}},\\ m_{\mathrm{Lout}}{\overset{..}{X}}_{\mathrm{Lout}}+c_{\mathrm{Lout}}{\overset{.}{X}}_{\mathrm{Lout}}+k_{\mathrm{Lout}}X_{\mathrm{Lout}}=F_{X_{\mathrm{Lin}}}-F_{Xr},\\ m_{\mathrm{Lout}}{\overset{..}{Y}}_{\mathrm{Lout}}+c_{\mathrm{Lout}}{\overset{\;.}{Y}}_{\mathrm{Lout}}+k_{\mathrm{Lout}}Y_{\mathrm{Lout}}=F_{Y_{\mathrm{Lout}}}+m_{\mathrm{Lout}}g-F_{Yr}. \end{array}\right.$$

where $F_{X_{\mathrm{Rin}}}$ and $F_{Y_{\mathrm{Rin}}}$ represent the bearing reactions of the inner race of bearing 1 in the X and Y directions, respectively, and $F_{X_{\mathrm{Rout}}}$ and $F_{Y_{\mathrm{Rout}}}$ correspond to the outer race, respectively. $F_{X_{\mathrm{Lin}}}$ and $F_{Y_{\mathrm{Lin}}}$ represent the bearing reactions of the inner race of bearing 2 in X and Y directions, respectively, and $F_{X_{\mathrm{Lout}}}$ and $F_{Y_{\mathrm{Lout}}}$ correspond to the outer race, respectively.

To clarify the influence of unknown time-varying noise on the dynamic model of bearings, the noise modules $F_{Xr}$ and $F_{Yr}$ denote components of external excitation $F_{r}$ in the X and Y directions, and $F_{r}$ is added to the dynamic formula, which is expressed as follows:

(15) $$F_{r}(t)=F_{r1}(t)+F_{r2}(t)$$

$F_{r1}(t)=F_{1}\delta_{1}(t)$ and $F_{r2}(t)=F_{2}\delta_{2}(t)$ are the forces generated by internal and external excitation acting on the rotor, respectively. $F_{r1}\left(t\right)$ and $F_{r2}\left(t\right)$ denote the zero mean Gaussian white noise, and are simultaneously satisfied with $E[F_{r1}(t)F_{r1}(t+\tau )]=2F_{1}\delta [t-\tau ]$ and $E[F_{r2}(t)F_{r2}(t+\tau )]=2F_{2}\delta [t-\tau ]$.

**Figure 3:**
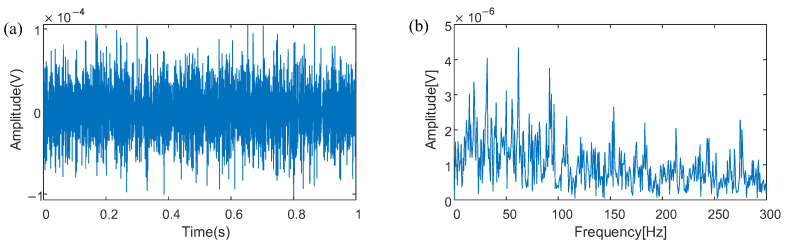
(**a**) Waveform of bearing signals from the X direction; (**b**) Spectra of bearing signals from the X direction.

To simulate the bearing outer ring fault signal submerged in intense background noise, Gaussian white noise alongside external excitations with forces of 0.7 N are subjected to the dynamic model of rolling bearings, to replicate complex and often chaotic conditions found in actual industrial environments, where multiple sources of vibration and noise can obscure diagnostic signals. The outcome of this simulation, illustrated in Figure 3, presents an analog signal that combines the outer ring bearing fault with significant background noise. It is evident from the signal that the distinct pulse components $\overset{..}{X}$, presumably related to bearing faults, are indiscernible within the noise. This highlights the challenge in identifying fault signatures when they are masked by environmental interference. In the analyzed envelope spectrum, the characteristic frequency associated with the rotation of power system components is evident, but specific frequency indicators of a bearing fault are not distinctly visible.

**Figure 4:**
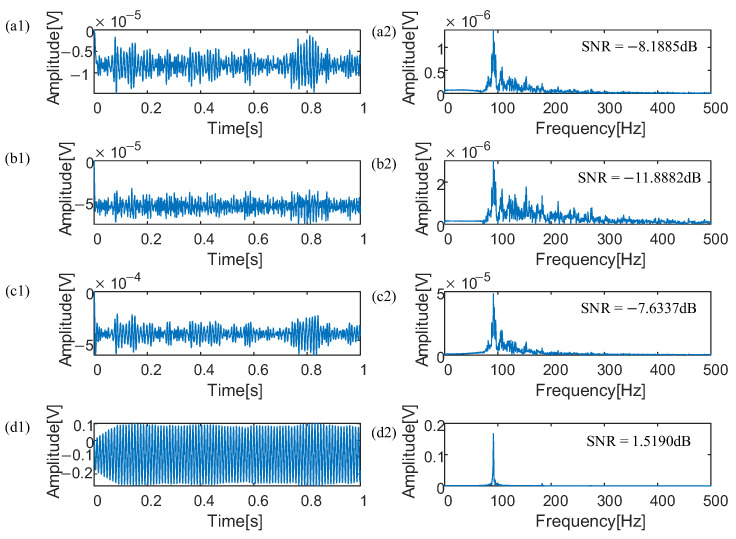
(1) Waveform of bearing signals processed by the SOSR method and the CDRN method; (2) Spectra of bearing signals processed by the SOSR method and the CDRN method.

To validate the efficacy of the proposed methodology, the contaminated envelope signal, which includes superimposed noise on the outer ring bearing fault signal, undergoes a noise reduction process. Subsequently, the optimized parameters for the model are autonomously derived through application of the DRL method optimizing coupling neurons as *h* = 0.1485, *Vth* = 0.1050, *a* = 0.7827, *b* = 0.6559, *D* = 0.5232, *V_re_* = 0.7486, *λ* = 0.6082, *W_f_* = 0.1518, *R* = 0.5391, and *δ* = 0.3936. The results of the optimal parameters are visualized in waveform and spectrum representations of the signals, which are illustrated in Figure 4d1,d2. Following application of the proposed method, the energy that was previously attributed to noise has been effectively reallocated to enhance useful components of the signal, and made the characteristic frequency of the bearing fault signal prominently distinguishable in the processed data. The SNR value is reported to be 1.519 dB, which indicates an improvement over the original signal and the utility of the DRL method optimizing coupling neurons in analysis of machinery health issues.

In the field of intelligent computing, QL (Q-Learning), PSO (particle swarm algorithm), and QPSO (quantum particle swarm algorithm) are three different types of methods, which are originated from reinforcement learning, population intelligence optimization, and quantum behavioral modeling, respectively. The core idea of QL is to learn the optimal behavioral strategies in the environment through a trial-and-error mechanism, and the intelligent agent performs the action in the state, updates the action value function (Q-value) according to the reward from the environment, and finally converges to the optimal strategy. The core idea of PSO is to simulate the group collaborative behavior of a flock of birds foraging for food. Each particle represents a candidate solution to the optimization problem, and achieves iterative optimization by tracking its own historical optimal position (individual extreme value, pbest) and the global optimal position shared by the group (global extreme value, gbest) to update its speed and position. The core idea of QPSO is to consider that the motions of the particles in PSO have quantum behavior, and to describe the probability distribution of a particle’s position through the quantum potential well model, so as to make the particles have a stronger all-around behavior in the search space with stronger global exploration ability and reduce the possibility of falling into local optimization.

To demonstrate the enhanced capabilities of the DRL method optimizing coupling neurons, the QL, PSO, and QPSO optimizing coupling neurons were applied on the simulated dynamic signal for extracting weak fault signals from noisy environments. The optimal parameters using QL optimizing coupling neurons are *h* = 0.0530, *Vth* = 0.3364, *a* = 0.7650, *b* = 0.4450, *D* = 0.2291, *V_re_* = 0.2064, *λ* = 0.5564, *W_f_* = 0.5732, *R* = 0.1, and *δ* = 0.4450, The optimal parameters using PSO optimizing coupling neurons are *h* = 0.1425, *Vth* = 0.4597, *a* = 0.9236, *b* = 0.2433, *D* = 1, *V_re_* = 0.8238, *λ* = 0.1514, *W_f_* = 0.7211, *R* = 0.139, and *δ* = 0.7036, The optimal parameters using QPSO optimizing coupling neurons are *h* = 0.2075, *Vth* = 0.4402, *a* = 0.3313, *b* = 0.3243, *D* = 0.1, *V_re_* = 0.1, *λ* = 0.1087, *W_f_* = 0.1, *R* = 0.527, and *δ* = 0.5227. Graphical representations illustrating the waveform of the signal and its spectral content following application of the QL optimizing coupling neurons are depicted in Figure 4a1,a2. The SNR is −8.1885 dB, which is reduced by 9.7075 dB when compared to outcomes achieved through the DRL method optimizing coupling neurons. Graphical representations illustrating the waveform of the signal and its spectral content following application of the PSO optimizing coupling neurons are depicted in Figure 4b1,b2. The SNR is −8.1885 dB, which is reduced by 13.407 dB when compared to outcomes achieved through the DRL method optimizing coupling neurons. Graphical representations illustrating the waveform of the signal and its spectral content following application of the QPSO optimizing coupling neurons are depicted in Figure 4c1,c2. The SNR is −7.6337 dB, which is reduced by 9.1527 dB when compared to outcomes achieved through the DRL method optimizing coupling neurons. Therefore, the method introduced in this manuscript not only facilitates the emergence of stochastic resonance even at minimal levels of vibration amplitude, but it also produces a distinct and recognizable characteristic frequency. This achievement enables an efficient and accurate identification of fault frequencies specifically associated with the outer rings of rolling bearings.

## 4. Applications

As critical components of mechanical equipment, bearings directly influence the operational stability and safety of machinery. Fault diagnosis enables the timely identification of potential faults, thereby preventing fault escalation and avoiding sudden equipment shutdowns or catastrophic accidents [65]. Through real-time monitoring and fault diagnosis of bearings, operational parameters and maintenance schedules can be optimized to ensure equipment operates under optimal conditions [66]. This not only enhances production efficiency and product quality but also reduces the probability of failures, thereby improving overall equipment reliability. Efficient bearing fault diagnosis and management help enterprises boost productivity, lower operational costs, and enhance product quality and market competitiveness [67]. Additionally, minimizing equipment failures and accidents safeguards employee safety and preserves corporate reputations. Therefore, it is imperative to develop and apply deep reinforcement learning–optimized coupled neuron models for bearing fault diagnosis, offering a robust solution to advance industrial reliability and safety standards.

**Figure 5:**
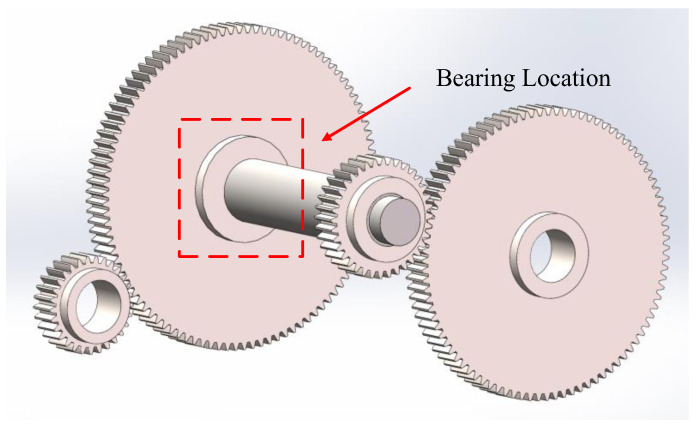
Schematic diagram of gearbox model.

The vibration signals of parallel gearbox bearings were experimentally analyzed, and the fault of the outer ring of the rolling bearing of the secondary parallel gearbox on the gearbox dynamics simulation test bench was experimentally studied. The model diagram of the gearbox is shown in Figure 5. The first and second gears form the first gear train and the third and fourth gears form the second gear train. The faulty bearing is located on the end cap of the third gear and the point of failure is located on the outer race of the bearing. The type of failure is pitting failure.

**Figure 6:**
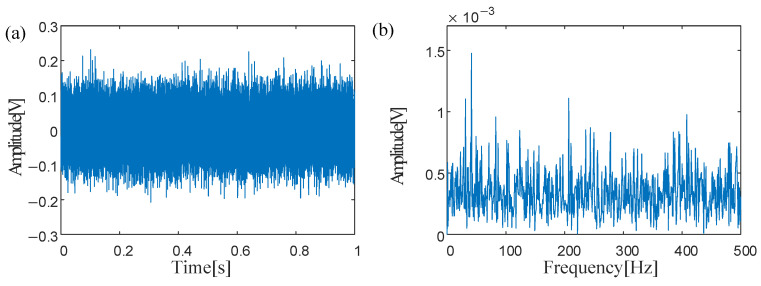
(**a**) Time-domain waveform of bearing faults; (**b**) Envelope spectrum of bearing faults.

In the experiment, the sampling frequency *f_s_* was set to 51.2 kHz, with 65,536 sampling points. The rotational frequency of the main bearing was 40 Hz. After speed reduction through the first gear stage, the second gear stage operates at a reduced speed, resulting in a bearing rotational frequency of 11.6 Hz. Vibration analysis revealed that the characteristic frequency of the outer race fault in the parallel gearbox bearing is *f_out_* = 41.04 Hz. Figure 6 depicts the time-domain waveform and envelope spectrum of the bearing fault. However, significant background noise obscures the characteristic frequency of the outer race fault in the gearbox rolling bearing, rendering it indistinguishable and preventing definitive diagnosis of the outer race fault.

**Figure 7:**
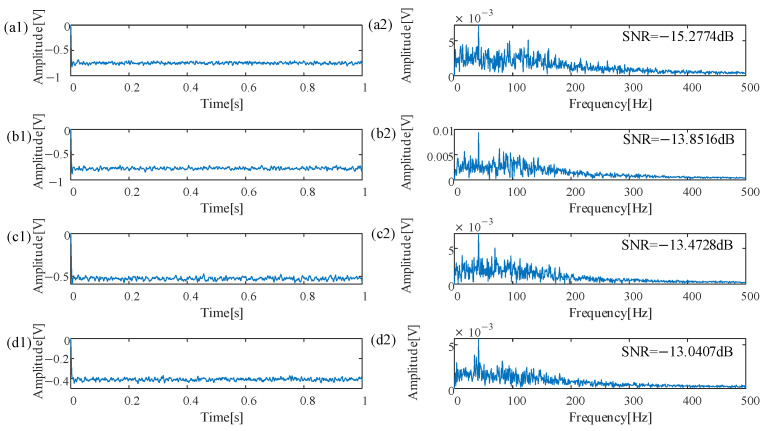
(1) Optimized time-domain waveforms; (2) Envelope spectra.

Parameter values are calculated by using a deep reinforcement learning algorithm to optimize the coupled neuron model and are *h* = 0.02, *Vth* = 0.9645, *a* = 0.7777, *b* = 0.6159, *D* = 0.52, *V_re_* = 0.7882, *λ* = 0.6214, *W_f_* = 0.8334, *R* = 0.5050, and *δ* = 0.5708. As shown in Figure 7d1,d2, the eigenfrequency 41.41 Hz is visible. This value is consistent with the theoretical value of 41.04 Hz from the signal from the parallel gearbox outer ring bearing, which indicates that the fault of the parallel gearbox outer ring has been correctly identified and verifies the effectiveness of the proposed method. The parameters of the coupled neuron model optimized using a reinforcement learning algorithm are *h* = 0.02, *Vth* = 0.7979, *a* = 0.8131, *b* = 0.5808, *D* = 0.8283, *V_re_* = 0.7677, *λ* = 0.6515, *W_f_* = 0.8081, *R* = 0.7475, and *δ* = 0.6819. The time-domain waveforms and envelope spectra are shown in Figure 7a1,a2. The signal-to-noise ratio at the eigenfrequency of the output signal is −15.2774 dB, which is reduced by 2.2367 dB compared with the method using deep reinforcement learning to optimize the coupled neuron model. The parameters of the optimized coupled neuron model using the particle swarm algorithm are *h* = 0.02, *Vth* = 0.5904, *a* = 0.6283, *b* = 0.6135, *D* = 0.7848, *V_re_* = 0.7303, *λ* = 0.8385, *W_f_* = 0.7277, *R* = 0.6437, and *δ* = 0.5133. The time-domain waveforms and envelope spectra are shown in Figure 7b1,b2, and the signal-to-noise ratio at the eigenfrequency of the output signal is −13.8516 dB, which is reduced by 0.8109 dB compared with the method using deep reinforcement learning to optimize the coupled neuron model. The parameters of the optimized coupled neuron model using the quantum particle swarm algorithm are *h* = 0.02, *Vth* = 0.6348, *a* = 0.6362, *b* = 0.5850, *D* = 0.53, *V_re_* = 0.5, _λ_ = 0.5017, *W_f_* = 0.5534, *R* = 0.5301, and *δ* = 0.5934. The time-domain waveform and envelope spectrum are shown in Figure 7c1,c2, and the signal-to-noise ratio of the output signal eigenfrequency is −13.4728 dB, which is reduced by 0.4321 dB compared with that of the method of optimizing the coupled neuron model with deep reinforcement learning. From the comparison of the above experimental data, it is clear that the optimization of the coupled neuron model using the deep reinforcement learning algorithm has superior performance in achieving energy enhancement of bearing fault features.

The intelligent identification method based on the optimization neural network is used to further verify the general applicability of the deep reinforcement learning algorithm in optimizing the coupled neuron model and thus extracting the bearing fault feature frequency, and the identification rate is used as an index to judge the ability of the reinforcement learning algorithm, particle swarm algorithm, quantum particle swarm algorithm, and deep reinforcement learning algorithm to optimize the coupled neurons to complete signal processing.

**Figure 8:**
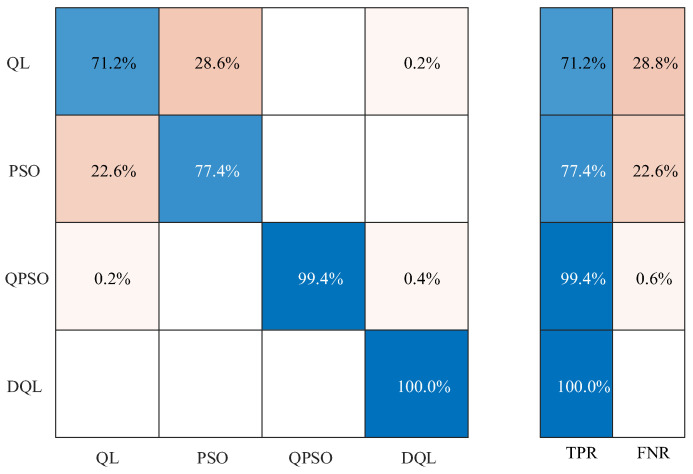
Fault recognition rate for each optimization algorithm.

Figure 8 shows the output signal further classified and recognized by using the artificial intelligence method based on a narrow neural network-based artificial intelligence method for further classification and identification of the output signal; it can be seen that the coupled neuron model optimized with the deep reinforcement learning algorithm has the highest fault identification rate of 100%. The fault identification rate of optimizing the coupled neuron model using the reinforcement learning algorithm is 71.2%, which is 28.8% lower than optimizing the coupled neuron model using the deep reinforcement learning algorithm. The fault identification rate of optimizing the coupled neuron model using the particle swarm algorithm is 77.4%, which is 22.6% lower than optimizing the coupled neuron model using the deep reinforcement learning algorithm, and the fault identification rate of optimizing the coupled neuron model using the quantum particle swarm algorithm is 99.4%, which is 0.6% lower compared to the optimized coupled neuron model using the deep reinforcement learning algorithm. The results of the bearing fault recognition rate data further indicate that the optimized coupled neuron model using the deep reinforcement learning algorithm has a high recognition ability in the diagnosis of weak bearing faults.

## 5. Conclusions

In this paper, a deep reinforcement learning optimization method based on noise processing is used. By optimizing the parameters in the coupled neurons, the best parameter combination with the lowest signal-to-noise ratio in the coupled neurons is obtained and applied to bearing fault detection. The effectiveness of this method is verified by comparison with reinforcement learning, particle swarm algorithm, and quantum particle swarm algorithm, and the following conclusions are obtained.

- Aiming at the problem of parameter optimization in coupled neurons, this paper proposes to use a deep reinforcement learning algorithm for optimization, so as to obtain the parameter combination with the best signal-to-noise ratio in coupled neurons, and apply it to bearing fault detection.
- An empirical playback region based on noise processing is introduced into the deep reinforcement learning framework, and the coupled neuron model parameter optimization algorithm driven by deep reinforcement learning is finally formed by filtering the playback region data with the signal-to-noise ratio as the optimization objective.
- Through experimental application of simulation signals and gearbox bearing fault vibration signals collected in a laboratory environment, the experimental results show that when the coupled neuron model is optimized by using the deep reinforcement learning algorithm, the signal-to-noise ratio of the output signal and the bearing fault recognition rate are −13.0407 dB and 100%, respectively, which are the best among the four comparison methods, verifying the effectiveness of the proposed method.

The research results of this paper not only have important engineering value in bearing fault diagnosis, but also provide new ideas and methods for fault diagnosis of other mechanical equipment. In the future, deep reinforcement learning algorithms can be optimized. For example, data in the experience playback area can be dynamically prioritized to replay samples that contribute more to model updates, and bearing diagnostic models can be further migrated to gearboxes, engines, and other equipment to improve the stability and safety of equipment operation.

## Data Availability

The data presented in this study are available on request from the corresponding author due to privacy.
